# From risky to safer home care: health care assistants striving to overcome a lack of training, supervision, and support

**DOI:** 10.3402/qhw.v8i0.20758

**Published:** 2013-05-23

**Authors:** Lena Swedberg, Eva Hammar Chiriac, Lena Törnkvist, Ingrid Hylander

**Affiliations:** 1 Department of Neurobiology, Care Sciences and Society, Centre of Family Medicine, Karolinska Institutet, Stockholm, Sweden; 2 Department of Behavioural Sciences and Learning, Linköping University, Linköping, Sweden

**Keywords:** Home care, home care technology, paraprofessional caregivers, training needs, grounded theory

## Abstract

Patients receiving home care are becoming increasingly dependent upon competent caregivers’ 24-h availability due to their substantial care needs, often with advanced care and home care technology included. In Sweden, care is often carried out by municipality-employed paraprofessionals such as health care assistants (HC assistants) with limited or no health care training, performing advanced care without formal training or support. The aim of this study was to investigate the work experience of the HC assistants and to explore how they manage when delivering 24-h home care to patients with substantial care needs. Grounded theory methodology involving multiple data sources comprising interviews with HC assistants (*n*=19) and field observations in patients’ homes was used to collect data and constant comparative analysis was used for analysis. The initial analysis revealed a number of barriers, *competence gap; trapped in the home setting; poor supervision* and *unconnected to the patient care system*, describing the risks associated with the situations of HC assistants working in home care, thus affecting their working conditions as well as the patient care. The core process identified was the HC assistants’ strivings to combine safe home care with good working conditions by using compensatory processes. The four identified compensatory processes were: *day-by-day learning; balancing relations with the patient; self-managing;* and *navigating the patient care system*. By actively employing the compensatory processes, the HC assistants could be said to adopt an *inclusive approach*, by compensating for their own barriers as well as those of their colleagues’ and taking overall responsibility for their workplace. In conclusion, the importance of supporting HC assistants in relation to their needs for training, supervision,and support from health care professionals must be addressed when organising 24-h home care to patients with substantial care needs in the future.

Patients receiving home care are becoming increasingly dependent upon competent caregivers around the clock due to substantial care needs, often including the use of home care technology (Midgren, 2007; National Board of Health and Welfare, 2008). In Sweden, these increased needs of advanced home care collide with the fact that caregivers are mostly paraprofessionals with limited or no health care training in performing advanced caring procedures. This raises questions regarding quality of care as well as accountability issues (National Board of Health and Welfare, 2005). Swedish county councils and municipalities are jointly responsible for the home care organisation. The county councils which are responsible for medical health care are largely divided into primary care and hospital care whereas the municipalities are responsible for long-term care and services in patients’ homes or in special accommodations such as nursing homes (Ministry of health and social affairs, 2007).

The caregivers in 24-h home care are commonly employed by the municipality to perform the one-to-one care on a daily basis, with or without being accompanied by family caregivers. On the request of the patient, a private staffing company may take over the service of caregiver employment and supervision from the municipality (National Board of Health and Welfare, 2005). In the literature, caregivers in home care are often described as paraprofessionals and defined as carers with limited health care training, not being members of a profession and working semi-autonomously under the direction from professionals (Kubiak & Sandberg, 2011; Wallach & Mueller, 2006). With increasing demands on advanced caring skills when “hospital care and home care is merging” (DePalma, 2008), paraprofessional caregivers’ working situation is becoming more challenging (Devlin & McIlfatrick, 2009; McIntyre, 2008). Lack of adequate training for the tasks (Laferriere, Bigelow, & Hallett, 1996; Nilsson, 2001), lack of control while working in a patients’ home (Soini &Välimäki, 2002; Vik & Eide, 2012), and low status and pay (Matthias & Benjamin, 2005) are already known factors. The caregivers’ situation is complicated due to the home setting as well as the organisational structures, which may result in stress-related problems and high turnover rates (Brulin, Winkvist, & Langendoen, 2000). Several studies concerning patient and family experiences on advanced home care points out insufficient caregiver competence as one of the most concerning issues (Ballangrud, Bogsti, & Johansson, 2009; Bjuresäter, Larsson, & Athlin, 2012). Inadequate systems for caregiver training may jeopardise patient safety (Ödegård, 2003). Home care technology, particularly when life-support home ventilators are involved, calls for caregiver training on technical knowledge as well as specific care procedures, such as airway clearance in order for caregivers to feel secure and satisfied (Ingadóttir & Jonsdóttir, 2006; Lindahl, 2010).

A recent study (Swedberg, Hammar Chiriac, Törnkvist, & Hylander, 2012), based on the same data sample as the present study, revealed that patients striving for safety in 24-h home care, selected certain caregivers to perform advanced procedures as well as instructed their new caregivers. However productive for the patients, these strategies seemed to create several challenges for the caregivers and could influence their work situation in a negative way. The caregivers in this Swedish study were paraprofessionals employed by the local municipality to provide 24-h basic and advanced care in patients’ own homes. The caregivers’ background varied and they held titles such as assistant nurse, carer, and personal assistant, but in this study the title *health care assistant* (HC assistant) will be used. Common to almost all HC assistants, was a lack of or very limited training in health care. *Basic care* in this study means assisting with personal hygiene, mobilisation, and nutrition as well as social company. *Advanced care* includes the assistance with patients’ specific care needs, such as administration of medication, tube feeding, and safe handling of home care technology in use. Some of the advanced procedures that the HC assistants performed were under the direction of health care professionals within the county council (National Board of Health and Welfare, 2006).

The overall aim of this study was to explore how the HC assistants, as paraprofessionals with limited health care training, managed their difficult tasks of caring for patients with substantial needs in their homes outside the security that comes with a hospital setting.

## Methods

Grounded theory methodology (GTM) was chosen because of the interest in generating theory and concepts about social processes in the participants’ natural setting, in this case interactions in the home care situation.There is a great variety of grounded theory methods (Bryant & Charmaz, 2007) most of which are overlapping and therefore we believe that the best way to describe the method used in this study is to be clear in describing the process of analysis (Hallberg, 2006). Our approach follows the principles of classic grounded theory (Glaser, 1978; Glaser & Strauss, 1967) with one important exception: we recognise the roots in symbolic interactionism (Blumer, 1986) and therefore rely on a constructivistic approach regarding ontology. Even if there is a clear intent to listen to the participants’ perspective, the researcher can never free him or her self from interpreting data. Thus, the main finding of a grounded theory study, which typically is a basic social process illustrated through a theoretical model, is regarded as a construction by the researcher in interaction with data (Blumer, 1986; Charmaz, 2005). The approach is in agreement with Hallberg (2006) and Guvå and Hylander (2003) and has been presented in several articles, for example, Swedberg et al. (2012), Hylander (2003), and Barimani and Hylander (2012).

### Participants and settings

The data source is described in Table I. A total of 19 HC assistants (17 female, 2 male) participated in the study; 12 were interviewed and observed and 7 were only observed. Their age varied between 20 and 53 (mean age=36.3 ). The participants were employed by the municipality and their educational background varied from a 3-year upper secondary school programme in Health and social care (*n*=3) regulated by the Swedish National Agency for Education (2010) to shorter courses and/or employer-supplied training (*n*=17). The shorter courses ranged from care of elderly to personal assistance while the employer-supplied training mostly consisted of work introductory days covering topics such as ergonomics and hygiene. The municipality nurse in charge delegated the administration of medication. When advanced home care technology was in use, such as a life-support ventilator, the hospital department responsible for the patient carried out training and delegation. However, due to a high turnover rate, several of the HC assistants did not have the above-mentioned training or delegations.

**Table I:** Data sources.

	Setting^1^	Patient characteristics		Health care assistants studied		
				
	No.	Age	Sex	Interviewed and observed (n)	Observed only (n)	Observation time (h)
	1	75	Female	1	3	20
	2	25	Male	4	4	30
	3	70	Female	4	0	20
	4	62	Male	3	0	8
Total	4			12	7	78

1 Home of patient.

The study settings were the homes of four patients in a medium-sized Swedish city. All patients were immobilised and dependent on the presence of one or two HC assistants around the clock, which resulted in workgroups of 8–17 HC assistants per patient. In addition to basic care, the patients needed advanced care, such as tube feeding, injections, and wound and stoma care. Home care technology included bed-lifts, electric wheelchairs as well as advanced home care technology such as feeding pumps, oxygen devices, and, in one setting, a life-support home ventilator. There were no next-of-kin living with the patients and none of the HC assistants was related to the patient. In addition to their local primary health care centres, the patients had several contacts with physicians within specialist care.

### Data collection

Data was collected by field observations and semi structured interviews (Bryman, 2012; Hammersly, 2007). A supervisor at the local municipality assisted with the enrolment of the patients. The lead author conducted the field study over a period of 2 years (2008–2009). Theoretical sampling was conducted continuously in accordance with GTM and the data collection was influenced by the on-going data analysis. The first sample (home setting 1) was selected to ensure several encounters with a variety of HC assistants. For subsequent sampling (home settings 2–4), these were selected to ensure variation in the type of care arrangement relating to factors such as patients’ age and care needs. The variation in data was optimised by visiting each patient on several occasions at different times of the day, which also enabled several HC assistants to be observed per home setting. In the final sample (home setting 4), the general observation time was reduced in favour of more specific observations and questions, which were guided by the emerging theory.

The lead author visited the patients’ homes frequently and participated in small talk to become familiar with the HC assistants, but did not become involved in care situations. The field observations were direct, with a low level of structure in terms of data gathering, which enabled the development of theory (Charmaz, 2005; Einarsson & Hammar Chiriac, 2002). Observations became more focused along with the development of the theory. Field notes were written during, as well as after the observation, and then transcribed into a total of 15 pages (approx. 4500 words). Qualitative face-to-face interviews with the HC assistants, 40–60 min in length, were conducted in accordance with a semi-structured interview guide (Table II). The questions became more focused as the analysis progressed and concepts and categories emerged. In addition to planned interviews, informal dialogues were conducted and reported in the fieldnotes (Kvale, 2008). Memos were written directly after each interview and during the analyses. The interviews were audio-taped, transcribed verbatim, and analysed before the next interview to identify important links between emerging codes.

**Table II:** Interview and observation guide.

Health care assistants were asked to describe experiences and feelings regarding
Patient's care needs
Relation to patients
Relation to colleagues
Own work situation
Contact with supervisors and health care professionals
Areas of improvement
Areas of observation
Care situations
Patient–health care assistant interactions
Health care assistant–health care assistant interactions

### Data analysis

The analysis began after the first field visit and continued concurrently with data collection. In the open coding, transcripts were read line-by-line and coded into 33 initial categories, for example, “get into the routines” and “one step ahead”. In the focused coding, categories were filled with new data; comparisons were made code-by-code and category-by-category. Similarities and differences were identified, and finally codes were condensed into four main categories with subcategories. Categories were conceptualised through theoretical coding and related to each other. Finally, data were coded against the basic social process and a theoretical model was developed to illustrate this social process. Findings were exemplified by quotes from HC assistants, complemented by notes from observations of caring situations. To assure variability in the data, quotes and notes from all home settings and informants were used.

### Ethical considerations

Information on the study was provided to all participants and written consent was obtained. Participant anonymity and confidentiality was guaranteed with special consideration taken regarding the patients enrolled in the study. Prior to sensitive care situations, patients were asked if the researcher (first author) had the permission to be present for observation purposes. Given the small sample size, confidentiality was maintained by not linking the quotes to specific informants and by using the pronoun “she” when quoting, regardless of the sex of the HC assistants or the patients. The study was approved by the Ethics Committee of the Karolinska Institutet, Stockholm (Dnr. 2007/170-31).

## Results

The HC assistants in the study delivered 24-h home care to patients with substantial care needs. They performed advanced care without formal training in home settings, making them exposed to great barriers affecting the patients as well as themselves. The HC assistants’ main concern was how to manage their working situation while experiencing barriers, identified in this study as *competence gap, trapped in the home setting, poor supervision* and *unconnected to the patient care system*. In their strivings to combine safe home care with good working conditions, the HC assistants were found compensating for the barriers, by means of *day-by-day learning, balancing relations with the patient*, *self-managing*, and *navigating the patient care system* (Table III).

**Table III:** Identified barriers and compensatory processes.

Identified barriers	Compensatory processes
**Competence gap**	**Day-by-day learning**
Lack of training	On-the-job-practice
No prior experience	Self-learning
Difficulties with learning in the workplace	Collegial learning
**Trapped in the home setting**	**Balancing relations with the patient**
Poor working environment	Staying sufficiently distant
Mastered by patient	Staying sufficiently close
**Poor supervision**	**Self-managing**
Lack of formal leadership	Forming informal leadership
Unclear routines	Peer-support
**Unconnected to the patient care system**	**Navigating the patient care system**
Lack of coordination of patient care	Coordinating patient care
Left alone	Acting one step ahead

### Competence gap (identified barrier)

#### Lack of training

The HC assistants expressed concern about the gap between what was expected of them and what they felt they could handle, “We are not trained for all the things they want us to do”. Working with a patient with a home ventilator constituted such a gap for one HC assistant who said, “I think this is strange, considering our patients’ care needs”. In spite of the fact that some courses in basic care were mandatory before employment, many HC assistants had not taken these courses and they expressed worries about their own as well as their colleagues’ lack of training.

#### No prior experience

Several of the HC assistants were young with little experience, which made their colleagues express concerns both about their ability to care for patients with substantial care needs and about their ability to perform ordinary house-hold work. *Difficulties with learning in the workplace* appeared when information about patients’ specific care needs was sparse. Also, it was difficult for new HC assistants to learn new techniques when patients preferred experienced HC assistants to undertake advanced care procedures. “It's difficult when I have to stand by and watch with no chance of hands-on practice”. Lack of time for reflection was yet another concern expressed by several of the interviewed HC assistants.

### Day-by-day learning (compensatory process for competence gap)

With their limited training, the HC assistants took responsibility in learning what they believed was needed in order to fulfil their tasks. The HC assistants compensated for their gaps in competence by seeking learning situations themselves through *on-the-job practice*. For example, when one patient only wanted experienced staff to shower her, a novice HC assistant came in on her day off to learn the procedure.

*Self-learning* was the major way to learn the routines and get used to the home care technology in order to achieve “full control and always knowing what to do”. This competence was observed when relaxed patients allowed the HC assistants to carry out complex tasks, such as transferring a patient from the bed to a chair using the electric hoist. Self-learning could be achieved by trouble-shooting, when the HC assistants experienced problems with the home care technology. One HC assistant described how she handled a stressful situation with a beeping and alarming home ventilator on a bus trip.

“When we finally got home I switched over to the spare ventilator and started to search for the cause of the problem. The alarms on the ventilator showed low pressure so I think it was a leakage. That can be really dangerous!”.

*Collegial learning* was a way to compensate for colleagues’ gaps in competence and to take responsibility for the overall level of competence in the workplace. All HC assistants needed to trust each other if something unexpected happened. New HC assistants usually attended a few introductory days with an experienced HC assistant. One junior HC assistant was observed following her senior colleague closely, letting the senior colleague decide both the order and pace of care for the patient. The experienced HC assistants passed on knowledge about their patient to new colleagues: “We tell them they need to be really careful. She is so fragile and we've seen her get seriously ill!”. In addition to teaching practical skills, the experienced HC assistants inspired new colleagues to adopt good work ethics, but also felt obliged to correct improper behaviour: “You don't lay down in the sofa with feet on the table while talking on the mobile phone for hours!”. Sometimes they checked up on the new HC assistants: “I don't want to spy or interfere, but I feel I need to know”.

### Trapped in the home setting (identified barrier)

The home setting put limits on care procedures and working conditions by the fact that it is the home of the patient and it is not built for advanced care situations. *Poor working environment* in the home setting was described as a barrier, which could put the HC assistants’ own physical and mental health at risk, for example: difficulty in manoeuvring patients in showers; working in homes in which the patient was a heavy smoker; and joint pain from heavy lifting. “I want to stay but don't know if I can”, said one HC assistant who suffered chest pain from the smoke.

Feeling *mastered by the patient* meant that HC assistants felt controlled in everything they did and said, which made it difficult to perform planned care. “She controls our time and can ask us if we're on vacation if we take just a short break”, one HC assistant described. This was also the case when patients asked them to change their work schedules, for example, to accompany the patient outside the house.

### Balancing relations with the patient (compensatory process for trapped in the home setting)

There was little that the HC assistants could do about a poor working environment, except alerting their supervisors. However, they could compensate for controlling patients by maintaining an appropriate balance in their relationship with the patient by acting professional without becoming too distant (*staying sufficiently distant*) and by being close but not too personal (*staying sufficiently close*). When the relationship was well balanced, the HC assistants described it as being in tune with the patient.

*Staying sufficiently distant*, meant that the HC assistants acted professionally, thus finding a balance between the patient's right to decide and her actual care needs. Some HC assistants liked the challenge of making the patient comply: “When she says no and I manage to persuade her in a good way, then I feel I have succeeded”. However, at other times, the HC assistants described that they need to stay “firm and strong”, because the patient did not appreciate how her own care needs could be met the best. HC assistants were also advised by their supervisors to support each other and sometimes set limits towards a patient. There was not always an agreement on the meaning of adequate professional distance. There were examples of HC assistants being too distant; for example, carrying out tube feeding at the same time as watching the television, without any interaction with the patient on the bed.

*Staying sufficiently close* meant that the HC assistant had a close relationship with the patient without becoming too personal. The HC assistants acknowledged that their workplace was also the patient's home: “We become very close because we are in her home, letting her decide as much as possible”. There was always a risk for the relationship to become too personal and when that happened it was criticised by colleagues. Sometimes the patient was involved in the selection of new HC assistants, and to be chosen was a positive factor. Often patients had favourites, typically among the experienced HC assistants, who were then willing to make personal sacrifices: “She doesn't want anyone but me, so I will work extra hours because of her”. Being a favourite could lead to disadvantages, such as diminished control over one's own working hours. Due to sudden diarrhoea, a difficult situation for junior HC assistants to handle, the patient asked the HC assistant to work on her day off and take a day's leave next week instead. HC assistants, who were not chosen, could feel less worthy compared to the favourites. One HC assistant described an occasion when her patient planned a trip with her “favourites”: “We were not asked if we wanted to come too. It felt hard not to be chosen”.

### Poor supervision (identified barrier)

*Lack of formal leadership* in their workplace was described by the HC assistants as a major barrier. Supervisors were rarely seen and contact was said to be sparse.

*Unclear routines* caused disagreements among the HC assistants. They described colleagues’ ignorance about existing routines, for example: “This workplace feels like a playground”. In the absence of a supervisor, HC assistants were concerned that duties that were not carried out correctly would never be discovered or corrected. The lack of clear routines meant that HC assistants felt abandoned when delegated the task of calling in extra staff. The 24-h care demanded good routines for staff scheduling and many concerns were associated with problems filling empty shifts.

### Self-managing (compensatory process for poor supervision)

When there was a lack of supervision, self-made workgroups were formed by the HC assistants characterised by informal leadership and peer-support. *Forming informal leadership* was one way to compensate for the lack of formal leadership. The HC assistants took over some of the supervisory role, such as maintaining contact with authorities and managing the staff schedule. On one occasion when shifts needed to be filled due to sick leave, the HC assistant was seen to stay on after her own shift and call in extra staff for the night. However, such informal leaders were not always accepted, and junior HC assistants complained that those who had worked for a long time in a given setting wanted to control everything themselves. *Peer-support* enabled the workgroup to become stronger and compensate for lack of routines. Good communication among the HC assistants enabled them to support one another, particularly if they felt threatened. For example, when questioned by the supervisor about the need for two HC assistants on a 24-h basis, the workgroup protested. “To be here alone would be a disaster” they said, referring to the patient's substantial care needs and requirement for life-saving equipment. They could also collectively refuse additional duties and responsibilities: “If the feeding-tube falls out, we do not re-insert it. We are not delegated to do that and therefore we refuse to do that”.

### Unconnected to the patient care system (identified barrier)

*Lack of coordination of patient care* obstructed HC assistants’ connection to their patient's professional care providers. They were described as being “on different planets”. “The patient's different doctors and nurses can't call or send an e-mail to each other”. Lack of coordination of care was also seen as a lack of interest: “The patient is so complicated, I don't think they want to learn more about her”.

*Being left alone*, was seen as a major risk and a barrier to safe care. Working alone was stressful to the HC assistants and particularly so when they experienced no support when calling for help. The HC assistants did not always know whom to contact or felt that they were not taken seriously when they did call for help. Calling when in need of help on a Friday evening, one HC assistant experienced that nobody answered in spite of the fact that she had been told to use this number, even on evenings and weekends. When in need of acute help, another HC assistant called 112 but was surprised to hear that they would not take the patient because the home ventilator required a special ambulance that was not available.

### Navigating the patient care system (compensatory process for being unconnected to the patient care system)

HC assistants compensated for being unconnected to the patient care system by navigating, which meant *coordinating patient care themselves* and *acting one step ahead*. HC assistants *coordinated patient care* themselves and were prepared to fight for patient rights. One HC assistant described how she had to beg for a home visit by the physician: “And when the doctor finally came, he stopped at the doorstep, totally startled when he realised how complicated the patient was”.

By *acting one step ahead* the HC assistants prepared themselves for emergencies, for example, by creating telephone lists and checking the first aid kit regularly. A patient outside the house accompanied with a life-support ventilator required an alert HC assistant: “I must have my eyes and ears alert all the time because bad things can happen to the patient anytime and anywhere, even on the bus!”.

### Summing up—the importance of an inclusive approach

By actively employing the compensatory processes, the HC assistants could be said to adopt an *inclusive approach*, by compensating for their own barriers as well as those of their colleagues’ and taking overall responsibility for their workplace. The HC assistants took responsibility for the overall level of competence through collegial learning and could balance their relationships with the patient in such a way that conflicts with the patient, as well as within the workgroup, were avoided. They also took on the roles as informal leaders and navigators. As a result, by acting inclusively, the HC assistants contributed to the overall effort to combine safe home care with good working conditions.

In contrast, some HC assistants were observed to compensate for their own barriers but not for those of their colleagues’. By not actively contributing to collegial learning, these HC assistants helped maintain a distinction between those who were competent and those who were not. Such an approach led to the risk of an HC assistant becoming a solo expert, with the attitude of “Things are not working if I'm not here”. Moreover, by accepting a role as a favourite of the patient, the balance in the relationship with the patient was disrupted and other HC assistants were excluded from the “inner circle”. In such a case, they did not take overall responsibility for the workplace. Therefore, by not acting inclusively, the HC assistants contributed less to the effort to combine safe home care and good working conditions.

## Discussion

The objective of this study was to investigate the work experience of the HC assistants and to explore how they manage when delivering 24-h home care to patients with substantial care needs, often including advanced care and home care technology. The study revealed the existence of several barriers experienced by the HC assistants in their roles as paraprofessionals with limited health care training as well as little support from supervisors and health care professionals. The study also revealed that despite the experienced and observed difficulties, HC assistants actively worked for a better outcome both for the patient and for themselves. One finding was that the HC assistants used strategies in order to maintain safe home care and good working conditions. The compensatory processes for different identified barriers were; *day-by-day learning, balancing relations with the patient*, *self-managing* and *navigating the patient care system*. An additional and important contribution was the identification of an *inclusive approach* among the HC assistants, describing their compensation for their own barriers as well as those of their colleagues’. The HC assistants then took responsibility for the overall situation by taking the learning needs of the colleagues into account, by balancing the relationship between the patient and workgroup and by taking on the role as an informal leader as well as a patient navigator.

### Focus on learning needs and future strategies is needed

The importance of competent caregivers in order to maintain safe patient home care is well documented (Ingadóttir & Jonsdóttir, 2006; Lindahl, 2010). In a previous study by (Swedberg et al., 2012), patients’ in 24-h home care, when experiencing a lack of competence among the HC assistants, actively selected competent ones and instructed the new HC assistants in their efforts to establish safe care. When the HC assistants in the present study felt they lacked competence, they took responsibility for self-learning, by using previous experience and by identifying self-learning situations themselves.

The described and observed learning situations, both self-learning and collegial-learning, took place in the actual setting, namely in the home in front of the patient and parallel with the daily care activities. This is well in line with how Lave and Wenger (1991) describe the characteristics of *situated learning*; Learning that takes place in the same function, context and culture in which it occurs (i.e., it is situated) and with social interactions and collaboration as essential components. The HC assistants’ individual drive to observe and learn was particularly observed when training needs were associated with the home care technology, also described by Brooks, Gibson, and DeMatteo (2008).

These strategies, searching situations of practical skill-training, corresponds to well-established concepts of learning, originating from Deweys’ (1933) “learning by doing”. Day-by-day learning, as seen in this present study, also fits well into Schons’ (1983) definition on “*reflection-in-action”*, sometimes described as “thinking on our feet”, including the use of own experiences and feelings in building new understandings of the situation that is unfolding. While reflection-in-action came naturally, the *reflection-on-action*, namely the activity that comes after a learning situation, was seldom seen or described by the studied HC assistants. The act of reflecting-on-action, i.e., talking things through with a supervisor or colleagues, enables us to spend time exploring why we acted as we did, what happened in the group, etc. (Schon, 1983). The wish for supervision as described by the studied HC assistants may reflect a lack of such reflection. The importance of reflection-on-action particularly concerning home care staff has been emphasised by others (Laferriere et al., 1996; Soini &Välimäki, 2002).

A lack of formal training made collegial learning necessary when new HC assistants were introduced to their work assignments. Collegial learning is similar to *peer teaching and peer learning*, commonly used in clinical education within nursing programmes (Secomb, 2008).

Through an inclusive approach as emphasised in this study, some of the HC assistants assumed the role of teaching and supporting novice HC assistants. Collegial learning may have positive effects on the competence level in the workplace. However, the risk of HC assistants instructing others with a low level of their own theoretical knowledge may be hazardous and therefore, the need for a minimum level of health care education is important to address. HC assistants in the study addressed a need for supervision. Their need could be fulfilled by organising reflection-on-action, led by an experienced person.

### Healthy relation to the patient is a key factor to success

The HC assistants described occasions when the patient's care needs collided with the patient's wishes, which made the situation challenging. Also there were occasions when the HC assistants’ working conditions collided with the patient's wishes. The HC assistants’ then tried to balance between actual care needs and patient's expressed needs as well as between their own working situation and the patient's wishes. The struggle to fulfill orders from supervisors, health care professionals as well as the wishes and needs of the patient in order to meet the needs of several parties is also outlined by others (Vik & Eides, 2012).

For the HC assistants, this became a balancing act between acting professional without becoming too distant and being close without becoming too personal in relation to the patient. When acting professional, the HC assistants mostly used coaxing to get the patient to accept care procedures. Occasionally, however, they felt obliged to use their authority to get their work done with the risk of overruling the rights of the patient. Having a care plan and rules to follow may help the HC assistants at times when the patient refuses to cooperate (Wallach & Mueller, 2006). The HC assistants in the study described their friendly relationships with the patients as very important. Sims-Gloud and Martin-Mattews (2010) also described building rapport, close connection, to the clients as one strategy used by paraprofessionals for the purpose of providing personalised care, for patient approval as well as for their own work satisfaction.

HC assistants maintained a balance in relation to the patient to counteract the blurring of professional boundaries. Mahmood and Martin-Matthews (2008), described it as “being in an intermediate position” when doing public work in a private domain. This was also experienced by registered nurses when expected to administer advanced “hospital care” in care settings for the elderly (Karlsson, Ekman & Fagerberg, 2009). Being chosen as a favourite was a special challenge to the HC assistant's balancing in relation to the patient, which could have both positive and negative consequences for the HC assistants. Although it was pleasing to be chosen, it created more demands and control from the patient, which contributed to blurring of the boundaries between work and private domains, for example when a patient controlled and changed the working hours of the HC assistants.

Favouritism also affected the work group in a negative way. Not being chosen as a favourite was described as rejection, which created a feeling of less worth and could lead to conflicts with the patient, as well as within the workgroup.

Although not easy to accomplish, the HC assistants with an inclusive approach did manage to balance the relationship with the patient as well as the workgroup, thus avoiding conflicts.

### Well managed workgroups are needed

The HC assistants often felt left alone with little support and in the absence of a supervisor, rules and routines were difficult to maintain and the workplace could even turn into a “playground”. The HC assistants themselves were forced to manage difficulties commonly connected to structures, roles, and processes of work groups (Brown, 2001; Hammar Chiriac, 2010).

The studied workgroups, which consisted of 8–17 HC assistants, seemed to compensate for these problems by employing *self-managing*, activities that may be understood using theoretical perspectives on *employeeship* (Bertlett, 2011). Employeeship includes co-operative relations between all employees, both leaders and followers. The lack of supervision and external support fostered a climate of *peer–employee style* among the HC assistants in contrast to a leader–follower style (Bertlett, 2011). When *peer-supporting*, the workgroups described herein, worked together to take control of their situation by strengthening their group and by setting limits toward the patient, supervisors and health care professionals. Peer support described within the health care context refers to initiatives when the relationship is one of equality and where colleagues give each other support (Dennis, 2003).

In the absence of a formal leader, informal leadership may develop if someone takes on the role and shows both psychological abilities to handle social interactions and required knowledge and skills to contribute in a given situation (Bertlett, 2011; Van Knippenberg & Hogg, 2007). In this study, experienced HC assistants who developed an inclusive approach, took on an informal leadership characterised by the above-mentioned abilities.

### The navigating role is important when chain-of-care is insufficient

Several HC assistants experienced a lack of coordination among the health care professionals, which affected both them and the patients. The importance of effective coordination for successful home care is well described by the Swedish national board of health and welfare (2005). The HC assistants in the present study navigated the patient care system by maintaining all contacts and coordinating patient care themselves. The assumption of the navigating role by patients and/or HC assistants, is supported by a previous study (Swedberg et al., 2012) where the patients’, when experiencing a poor continuity in their care, felt the need to navigate, either by themselves or by a competent HC assistant.

By being one step ahead, HC assistants were prepared for emergencies, because they were unsure of how to obtain support when in need of help. The degree of stress could be extremely high when no help was received even when they called 112, which echoes the findings of Gysels and Higginson (2009), who studied caregiver stressors in palliative home care. The aim of the observed navigating strategies, which to the best of our knowledge has not been described previously, was to avoid such situations of extreme stress.

## Summary

Our study shows that the HC assistants often perform advanced care in home settings without formal training or support. They strive to combine safe home care with good working conditions using strategies to compensate for the experienced barriers. In order to succeed, the need for HC assistants to assume an inclusive approach has been identified. HC assistants with an inclusive approach facilitate day-by-day learning, balance the relations with the patient, take responsibility for self-managing and navigate the patient care system, thereby taking responsibility for the workplace overall. The study data indicated work experience as an important factor among HC assistants with an inclusive approach.

The risks of high turnover rates, as well as the importance of continuity in home care, are well known and the importance of preventing a future shortage of paraprofessionals in health care is stressed in the literature (Woodward, Abelson, Tedford, & Hutchinson, 2004). The overall working conditions are essential and need to be taken seriously. The level of health care knowledge and empowerment are both key factors for increased job satisfaction and the possibility for paraprofessionals to stay in home health care (Johnson & Noel, 2007). Important factors for achieving empowerment include structured role assignments, participation in decision-making, positive relationships with managers and collaborative peer relations (Wallach & Mueller, 2006). In addition to the challenge of recruiting and retaining HC assistants to meet the increasing need of home care in the future, the issue concerning patients with substantial care needs must be addressed.

In Sweden, the consequences of using paraprofessionals with limited health care training in 24-h home care, involving advanced procedures and technology need to be further evaluated. Collegial learning, i.e., learning from colleagues and daily-situated learning were the main strategies to overcome a lack of competence. Furthermore, experienced HC assistants were found to be very important to their less experienced colleagues. Thus, structured situated learning using experienced HC assistants as supervisors may be one way to overcome the competence gap. In addition, there was a need not only for situated learning but also for reflection on action. To serve as supervisor in that role and to guarantee updated care competence, further training for experienced HC assistants is called for, which could also act as an incentive for experienced HC assistants to stay in their position.

## Strengths and limitations

The strength of the present study was that data were collected from interviews as well as observations. One limitation is that the presence of the lead author, a registered nurse, might have influenced the HC assistants to give a more positive representation of their part in the care. However, the authenticity of the situations observed is supported by the fact that both positive and less positive care situations were observed. In addition, the lead author has worked in close collaboration with the other authors, who are from different disciplines, to enhance the credibility (Bryman, 2012) of the study.

Rigour was ensured by immediate transcription of the observational data, in addition to memo writing. Although grounded in empirical data, the theoretical model that resulted from the present study should be regarded as a set of proposals. It is a substantive theory that is applicable to the context from which it emerged, i.e., HC assistants working in 24-h home care in a medium-sized Swedish city. However, it might also have relevance for similar situations and contexts (Larsson, 2009).

## Conclusion

The HC assistants in this Swedish study compensated for their lack of training and support from supervisors as well as health care professionals by using strategies to ensure patient safety and their own working conditions. The substantial care needs among the patients resulted in a home care somewhat close to hospital care and as paraprofessionals, they were left alone with responsibilities that far exceed their level of training. In Sweden, the consequences of using paraprofessionals with limited health care training in 24-h home care, involving advanced care and home care technology, needs to be further evaluated regarding the need for minimum standards for training, supervision and support. According to our model, HC assistants with abilities to take overall responsibility for the workplace was found to be very important, thus making the care as safe as possible regardless of the level of training and support from the health care system. This finding underscores the importance of ensuring that experienced HC assistants who are well suited for their work are provided with working conditions and incentives that make them stay in their position.
